# Early Experience with Customized, Meal-Triggered Gastric Electrical Stimulation in Obese Patients

**DOI:** 10.1007/s11695-014-1498-1

**Published:** 2014-11-15

**Authors:** M. Miras, M. Serrano, C. Durán, C. Valiño, S. Canton

**Affiliations:** Obesity and Laparoscopy Unit, Clínica La Luz, General Rodrigo 8, 28003 Madrid, Spain

**Keywords:** Obesity, Gastric stimulation, Gastric stimulator, Weight loss

## Abstract

**Background:**

We report our initial gastric electrical stimulation experience using the abiliti® system for the treatment of obese patients followed for 1 year.

**Method:**

Between March 2011 and June 2013, 27 obese patients (BMI 30 to 46 kg/m^2^) were enrolled in a prospective open label study and implanted with a gastric stimulator. The patients were provided with nutritional support, and sensor-based behavioral feedback.

**Results:**

At 12 months, percent excess weight loss (%EWL) obtained was 49.3 ± 19.2 % with no significant differences between gender or age sub-groups. The %EWL data were segmented into two groups according to BMI 30–40 kg/m^2^ patients (obesity grade I and II) and BMI >40 kg/m^2^, with the results of weight loss being significantly higher for the lower BMI group (59.1 ± 19.5 vs. 46.7 ± 13.4, respectively, *p* < 0.01). One subject requested to have his device explanted, and the minor postoperative adverse events were resolved without hospital admission. All patients experienced early satiety and reduced their intake.

**Conclusions:**

After 12 months of follow-up, gastric electrical stimulation treatment appears to be a safe and effective option for weight loss in obese subjects. Long-term follow-up and further studies are warranted.

## Introduction

The prevalence of obesity continues to increase [1], the latest data from Spain show that 24.4 % of the adult male and 21.4 % of female are obese [2]. Bariatric surgery has proven effective, but is accompanied with morbidity (e.g., leakage, infection) and side effects. For these reasons, less invasive solutions for the obesity treatment are sought, providing satisfactory results. One of them is gastric electrical stimulation (GES) which produces an early feeling of fullness in order to reduce food intake.

The abiliti® system (IntraPace Inc) consists of a *lead* with two electrodes: a transgastric sensor detecting food intake and a stimulation electrode placed at the lesser curvature over the vagus. This lead is connected to a *stimulator* that sends electrical impulses when intake is detected. The system includes a 3D accelerometer recording physical activity, and an intragastric food sensor recording daily intake, providing objective data to the medical team. A telemetry link enables data download for patient monitoring.

The aim of this prospective open label study was to evaluate the effectiveness of GES on weight loss in a patient population unwilling to undergo to more invasive bariatric surgery. The study was approved by the hospital ethics committee and conducted in accordance with Good Clinical Practice and consistent with the Declaration of Helsinki. The patients signed informed consent prior to their participation and supported the financial costs of their procedures with no IntraPace financial support provided for either the patient or clinician.

The devices were implanted in the Obesity Surgery Unit of our hospital, during the period between March 2011 and June 2013. We present our experience at 12 months post-surgery.

## Patients and Methods

Twenty-seven adults with a BMI in the class I or II obesity range (World Health Organization) [3] or class III range unwilling to undergo a bariatric surgery were proposed the GES treatment alternative.

Patients were required to be >20 years of age, with no previous of gastric surgery, and willing to comply with the follow-up schedule, post-procedure diet, and exercise program. Multidisciplinary interviews with the medical and surgical teams, baseline lab measurements including HbA1c, and completion of the Three Factors Eating Questionnaire (TFEQ) [4] which characterizes the patient’s eating behavior, were reviewed prior to the surgical decision. GES efficacy was evaluated using weight loss, expressed as percent excess weight loss (%EWL), with expectations set at a minimum of 35 % at 12 months. Exploratory analysis on the effects of gender or baseline BMI on weight loss outcome was performed. Safety data were collected and analyzed during the study period.

### Device Implant Technique

**Fig. 1:**
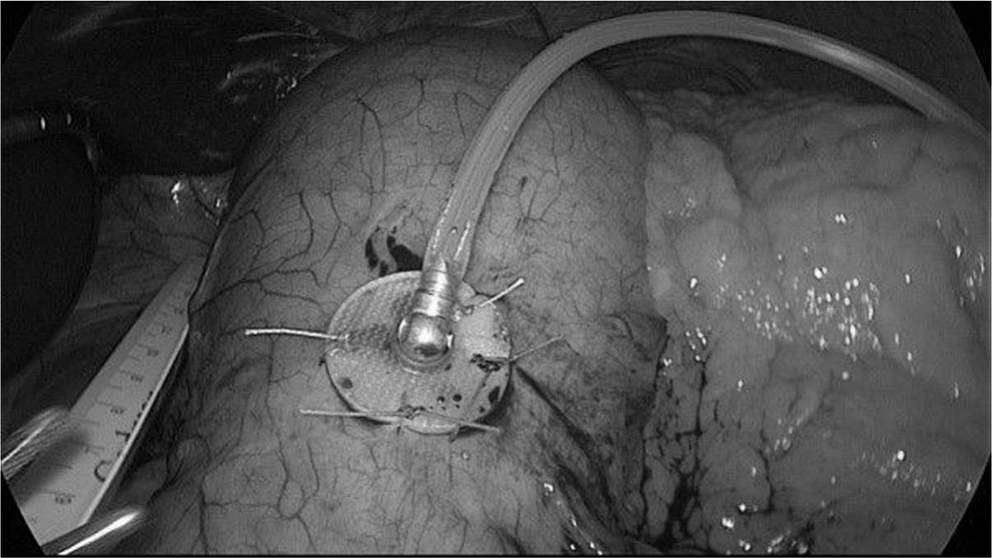
The transgastric food sensor is secured to the gastric serosa by suturing the silicon flange

The implant is performed using standard laparoscopic technique with the usual preoperative protocol for bariatric surgery. The patient was placed in lithotomy position; a pneumoperitoneum is created by Veress needle puncture to an intra-abdominal pressure of 14 mmHg. Three abdominal trocars were placed, at midline supra-umbilical for optical 5th, in the right upper quadrant, and in the left subcostal region (anterior axillary line). The anterior wall of the stomach was explored to identify the ideal site for the electrodes placement. The food sensor was inserted in the body-fundus region, about 3 cm from the greater curvature. The stimulation electrode was sutured approximately 4 cm from the gastroesophageal junction and 1.5 cm from the lesser curvature, over the division branches of the Latarjet nerve (*goose’s foot*). The distance between electrodes was 3–4 cm. Upon inflation of the stomach, a dilating needle is inserted to create an opening for the insertion of the sensor probe; the silicon flange is then secured to the serosa, to prevent the electrode receding through the gastric conduit in the peritoneal cavity (Fig. 1).

**Fig. 2:**
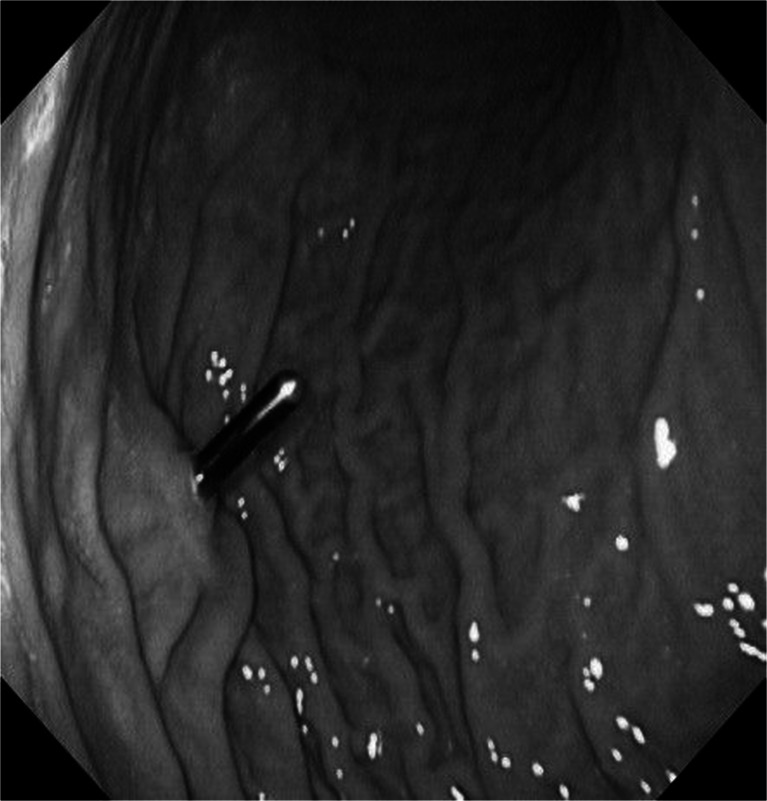
Intragastric extension of the food sensor probe

An endoscopic exam confirmed the intragastric position of the sensor probe and its extension into the gastric lumen (10–11 mm) (Fig. 2).

**Fig. 3:**
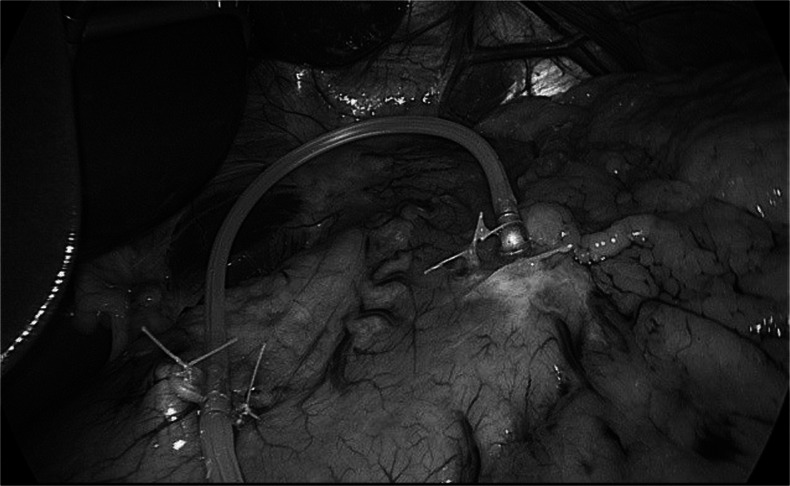
The stimulation electrode is secured to the serosa over the vagus nerve branches

The stimulating electrode is placed flush to the stomach surface over the vagus branches and secured to the serosa. It is important to verify that the lead length between the sensor and stimulation electrode is adequate to accommodate the gastric distension (Fig. 3).

The proximal end of the lead is externalized to the subcutaneous pocket created to fit the stimulator. The two lead probes are inserted and secured in the stimulator ports, and the excess lead is coiled under the stimulator for protection; the pocket is then closed with absorbable sutures.

### Post-Implant Follow-up

All the patients were discharged 12–24 h after surgery with liquid or soft diet for 48 h. Their devices were programmed to deliver a mild stimulation at mealtimes. One week post-surgery, the patients attend a consultation for surgical site evaluation and tailoring programmed stimulation. The patients are seen monthly for the first 3 months post-surgery then, every 2 to 12 months. At each visit, nutritional plan and physical activity guidelines were reviewed and stimulation parameters were adjusted if needed.

### Statistical Analyses

Baseline subject characteristics were summarized using mean ± SD for continuous variables and *n* and percent for categorical variables. Excess weight loss (EWL) was calculated using the measured weight minus ideal weight, determined according to the WHO recommended healthy BMI of 25. Sub-group analysis was done using the Student *t* test for statistical significance to determine the association of BMI, gender, and age with %EWL at 1 year.

## Results

Twenty seven obese subjects with no associated comorbidities underwent the device implant. One subject requested her device explanted after 2 months and is not included in this analysis. Nine men and seventeen women completed the 12-month follow-up. The baseline characteristics are reported in Table 1.

**Table 1:** Baseline demographics (mean ± SD (range)), all subject implanted

Gender	Female, *n* = 18 (66.6 %), Male, *n* = 9 (33.4 %)
Age (years) mean ± SD (range)	41.2 ± 9.2 (28–61)
BMI (kg/m^2^) mean ± SD (range)	40.0 ± 5.7 (30–60)
Obesity grade I	*n* = 4 (14.8 %)
Obesity grade II	*n* = 9 (33.3 %)
Obesity grade III	*n* = 14 (51.9 %)

### Efficacy

**Fig. 4:**
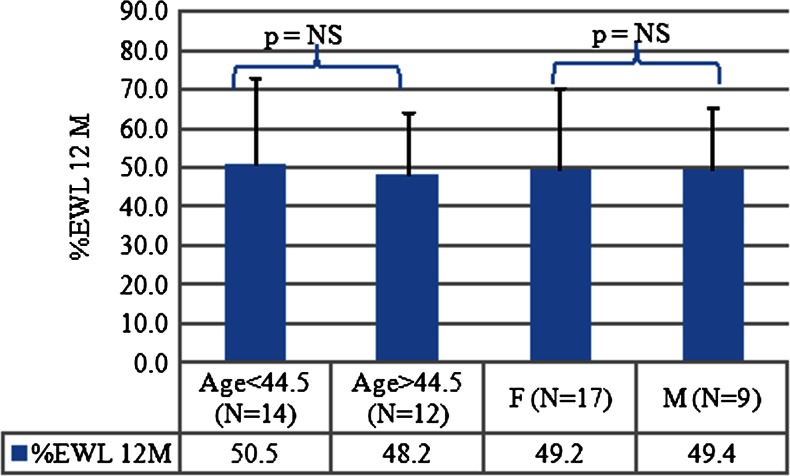
%EWL difference between gender males (*n* = 9) and females (*n* = 17), or age group <44.5 years old (*n* = 14) and >44.5 years old (*n* = 12)

At 12 months, the %EWL, percent weight loss (%WL) and weight loss for all the subjects were 49.3 ± 19.2 %, 17.0 ± 5.0 %, and 19.1 ± 6.5 kg (mean ± SD), respectively. No significant difference in %EWL was found between gender (9 M, 17 F) 49.4 ± 15.8 % vs. 49.2 ± 21.2 %, respectively, or age <45 years (*n* = 14) vs. ≥45 years (*n* = 12), 50.5 ± 22.5 % vs. 48.2 ± 15.9 % (mean ± SD), respectively (Fig. 4).

**Fig. 5:**
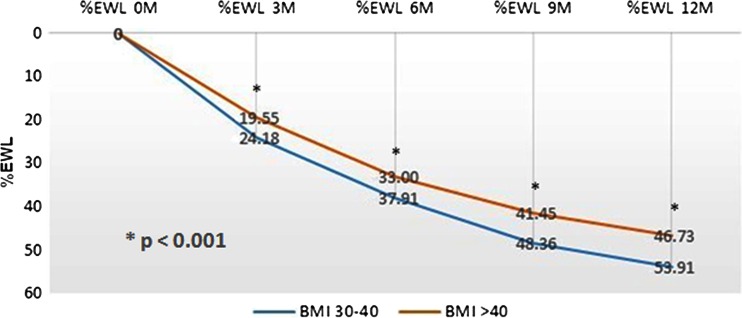
% Excess weight loss at 3, 6, 9, and 12 months for subjects with BMI 30–40 m^2^/kg (*n* = 13) *blue line* and with BMI >40 m^2^/kg (*n* = 13) *red line*

**Fig. 6:**
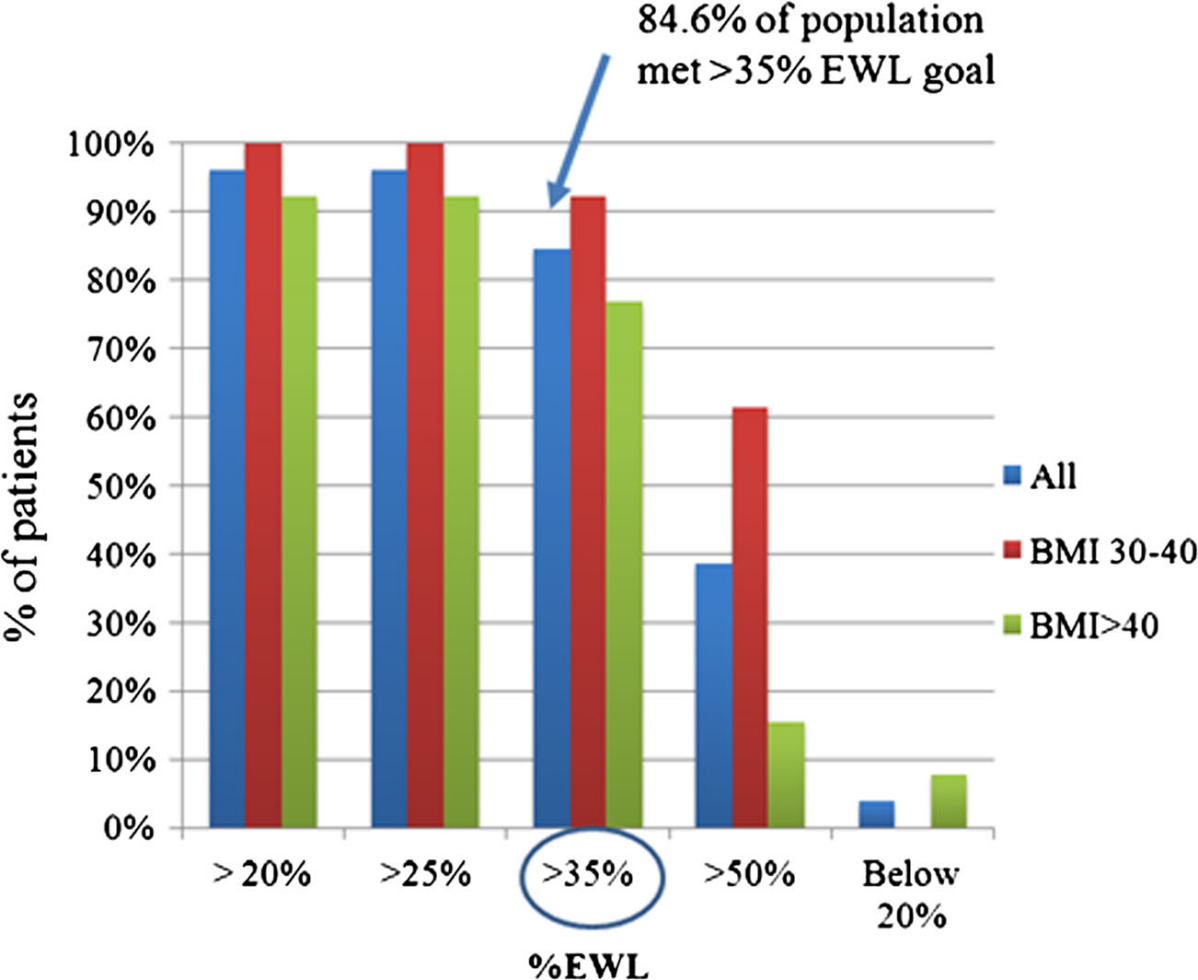
Percentage of patients that achieved different %EWL milestones at 12 months. *Blue column* total population (*n* = 26), *red column* patients with BMI 30–40 m^2^/kg (*n* = 13), *green column* patients with BMI >40 m^2^/kg (*n* = 13). Greater than 80 % of the population met the study goal of >35 % EWL

A sub-analysis according to the baseline BMI was performed—BMI 30–40 (*n* = 13) and BMI >40 (*n* = 13). At 12 months, the analysis show a significant difference between lower and higher BMI groups in %EWL (%EWL 59.1 ± 19.5 vs. 46.7 ± 13.4, respectively, *p* < 0.01). The %EWL calculated at 3, 6, 9, and 12 months are presented in Fig. 5 and Table 2. In both groups, our minimal goal of 35 % was exceeded; 22 subjects (84.6 %) obtained a %EWL > 35 % at 12 months (Fig. 6).

**Table 2:** Mean percent excess weight loss at 3, 6, 9, and 12 months post-implant

% EWL mean ± SD (range) (months)	All subjects (*n* = 26)	BMI 30–40 Kg/m^2^ (*n* = 13)	BMI >40 Kg/m^2^ (*n* = 13)
3	21.8 ± 10.5 (7–51)	27.1 ± 11.5 (11–51)	19.5 ± 6.1 (7–26)
6	35.3 ± 14.7 (14–70)	42.8 ± 16.0 (20–70)	33.0 ± 8.7 (14–44)
9	44.2 ± 17.3 (13–82)	53.2 ± 17.5 (37–81)	41.5 ± 11.6 (13–61)
12	49.3 ± 19.2 (10–90)	59.1 ± 19.5 (34–90)	46.7 ± 13.4 (10–68)

### Safety

The average duration of surgery was 50.1 ± 11.0 min (range 37–73) without any intra-operative complication. There was no death; all patients were discharged 12–24 h post-surgery. Two subjects presented a mild fever in immediate post-surgery with no clinical or surgical diagnosis evident. The fever receded with oral antibiotic and did not delay the patient hospital discharge. One patient requested her device to be explanted after 2 months stating that she did not achieve her desired weight loss. The device was explanted under a laparoscopic procedure.

Three patients presented with mild stomach cramping during the stimulation period, these symptoms disappeared with stimulation reprogramming. There was no other therapy side effects reported (e.g., nausea, vomiting) during the study period.

## Discussion

GES with abiliti produced an early sensation of fullness and satiety as reported by the patients, helping to reduce their food intakes. The surgical procedure is minimally invasive with minor complications in comparison with other bariatric surgeries.

The GES Transcend® system showed an efficacy ranging from 21 to 23 % EWL at 12 months [5–9], the Tantalus-Diamond® (Metacure) designed for contractility modulatory therapy improved glycaemic control and caused a modest weight loss in patients with Type 2 diabetes 5.4 ± 1.6 %WL at 12 months [10], and most recently, VBLOC (EnteroMedics), a system designed for a non-selective block of vagus activity, obtained 25 % EWL at 12 months [11]. The location and parameters of the abiliti® are similar to the Transcend, and studies have shown that this type of stimulation food intake reduction is probably driven by direct and indirect activation of afferent neural pathways in the vagus nerve that produce a satiety response [12–14]. Improved efficacy of the abiliti may be related to more selective activation of vagal afferents through patient-tailored stimulation. Also, the “sensor intake dependant” stimulation versus continuous stimulation reduces significantly the neuromuscular adaptation phenomenon [15] probably responsible for loss of efficacy. Finally, providing stimulation above a subject’s symptomatic threshold supports conscious modification of eating behavior.

In addition to food intake, physical activity is detected by the device 24 h a day; these data may be discussed with the patient, providing behavioral feedback, helping to correct efficiently the patient’s lifestyle. Combining the effect of gastric stimulation with behavioral management likely leads to more significant weight loss results.

The minimum target of 35 % EWL was chosen based on the weight loss efficacy range observed with gastric banding [16]. The range of WL achieved in our population was wide (10 to 90 % EWL with a mean of 49.3 %); however, only two patients achieved less than 30 % EWL, and both had a BMI >40 at baseline. We hypothesize that initial patient’s screening based on physiological or psychological characteristics and eating behavior profile would lead to better results. The average of WL 19.1 ± 6.5 kg or 17.0 ± 5.0 % achieved exceeded the weight loss shown to result in meaningful reduction in mortality and comorbidities [17–19]; the authors conclude that moderate weight loss of 10 % results in a 25 % reduction in overall mortality, 40 % reduction in diabetes-related deaths, and 50 % reduction in obesity-related malignancy and also reduced the incidence of hypertension, hypercholesterolemia, and type 2 diabetes.

## Conclusions

Gastric electrical vagal neuro-modulation is a valuable option to treat obesity. The results obtained are similar to restrictive procedures. GES creates early sensation of fullness and food intake reduction to obtain significant weight loss. The information tracked by the food sensor and the accelerometer help physicians in obesity management programs. This therapy facilitates and enhances the behavior monitoring programs developed in our hospital. Studying more patients will increase our expertise in gastric neurostimulation and help define the most responsive population. Possible mechanisms of action of this therapy remain to be defined. These results need to be confirmed with additional clinical studies.
